# Efficient *In Vivo* Screening Method for the Identification of C_4_ Photosynthesis Inhibitors Based on Cell Suspensions of the Single-Cell C_4_ Plant *Bienertia sinuspersici*

**DOI:** 10.3389/fpls.2019.01350

**Published:** 2019-10-30

**Authors:** Alexander Minges, Dominik Janßen, Sascha Offermann, Georg Groth

**Affiliations:** ^1^Cluster of Excellence on Plant Sciences (CEPLAS), Institute of Biochemical Plant Physiology, Heinrich Heine University, Düsseldorf, Germany; ^2^Institute of Botany, Leibniz University, Hannover, Germany

**Keywords:** C_4_ photosynthesis, Inhibitor, phosphoenolpyruvate carboxylase, pyruvate phosphate dikinase, *Bienertia sinuspersici*, cell suspension, screening

## Abstract

The identification of novel herbicides is of crucial importance to modern agriculture. We developed an efficient *in vivo* assay based on oxygen evolution measurements using suspensions of chlorenchyma cells isolated from the single-cell C_4_ plant *Bienertia sinuspersici* to identify and characterize inhibitors of C_4_ photosynthesis. This novel approach fills the gap between conventional *in vitro* assays for inhibitors targeting C_4 _key enzymes and *in vivo* experiments on whole plants. The assay addresses inhibition of the target enzymes in a plant context thereby taking care of any reduced target inhibition due to metabolization or inadequate uptake of small molecule inhibitors across plant cell walls and membranes. Known small molecule inhibitors targeting C_4_ photosynthesis were used to validate the approach. To this end, we tested pyruvate phosphate dikinase inhibitor bisindolylmaleimide IV and phosphoenolpyruvate carboxylase inhibitor okanin. Both inhibitors show inhibition of plant photosynthesis at half-maximal inhibitory concentrations in the sub-mM range and confirm their potential to act as a new class of C_4_ selective inhibitors.

## Introduction

A number of different types of photosynthesis — C_3_, C_4_, and crassulacean acid metabolism (CAM) — are known for terrestrial plants. Each of them is specialized to accommodate different environmental conditions with C_3_ photosynthesis being the most common one. CAM and C_4_ plants adapted to hot and dry climate by establishing CO_2_ concentrating mechanisms that circumvent the oxygenase reactivity of ribulose-1,5-bisphosphate carboxylase/oxygenase (RuBisCO) which is the key enzyme of photosynthetic carbon fixation within the Calvin-Benson-Bassham (CBB) cycle. In C_4_ plants, this CO_2_ concentrating mechanism is implemented by a spatial separation of CO_2_ uptake from the CBB cycle. Normally, this separation is established across two cell types: bundle sheath (BS) cells which are surrounded by a layer of leaf mesophyll (LM) cells forming the so-called Kranz anatomy. Primary CO_2_ fixation takes place in the LM cells, where atmospheric CO_2_ is fixed after conversion to HCO_3_^−^ by phosphoenolpyruvate carboxylase (PEPC) yielding the four-carbon organic acid oxaloacetate that is subsequently converted species-specifically into malate or aspartate. These diffuse to the BS cells where CO_2_ is released by decarboxylation and hereby enriched within the BS cells. The local increase in CO_2_ concentration greatly disfavors the oxygenase activity of RuBisCO which then uses the released CO_2_ during the CBB cycle to form triose phosphates. Pyruvate formed in the process of decarboxylation diffuses back into the LM cells and is regenerated to phosphoenolpyruvate (PEP) by the pyruvate phosphate dikinase (PPDK). The regenerated PEP then may be reused to fix another molecule of CO_2_. With the discovery of plants capable of performing single-cell C_4_ photosynthesis (SCC_4_), it has been shown however that Kranz anatomy is not a mandatory requirement for C_4_ photosynthesis (Sage, 2002; Voznesenskaya et al., 2002).

Many important crop plants like soybean, sugar beet or wheat are C_3_ plants, while some of the most abundant weeds such *Amaranthus retroflexus* or *Echinochloa crus-galli* utilize C_4_ photosynthesis. With the rise of known resistances of weeds to commercially available herbicides over the past decades the effects of resistant weeds on modern agriculture are becoming an increasingly pressing issue (Heap, 2014; Busi et al., 2018; Heap, 2019). In addition to herbicide resistances, the predicted increase in average temperatures caused by global warming and rising levels of atmospheric CO_2_ are thought to shift the odds in favor of C_4_ plants which will eventually render C_4_ weeds even more competitive in the context of C_3_ crops (Fernando et al., 2016; Carboni et al., 2017; Waryszak et al., 2018). To combat this development, new herbicides that are specifically designed to target C_4_ weeds are needed.

The identification and characterization of novel inhibitors interfering with mandatory biochemical pathways in plants are key requirements for the development of new herbicides. This process usually includes *in vitro* assays which assess the efficiency and efficacy toward a specific target enzyme followed by extensive *in vivo* studies using whole plants. However, compounds that prove to be effective *in vitro* may not be effective at all on whole plants due to reduced bioavailability which might be caused by several issues such as slow uptake into the plant tissue, solubility problems or detoxification of the active compound within the cells (Shimabukuro, 1985). Furthermore, *in vivo* studies with whole plants are cumbersome — particularly in terms of set-up and repetitions, human resources, and technical facilities required for a controlled environment. Consequently, an alternative *in vivo* method for the preliminary validation of biological effectiveness of compounds is needed for pre-screening purposes.

In recent years, a number of inhibitory compounds targeting C_4_ photosynthesis have been identified (Haines et al., 2005; Nguyen et al., 2016; Dick et al., 2017; Minges and Groth, 2017). These compounds inhibit PPDK or PEPC activity, which are key enzymes in Kranz C_4_ and SCC_4_ pathways. Effects of C_4_ inhibitors have been previously studied *in vitro* using purified enzymes in spectrophotometric assays (Doyle et al., 2005; Motti et al., 2007; Nguyen et al., 2016; Minges and Groth, 2017). While *in vitro* experiments are usually fast and convenient to perform and scale-up well (Feng et al., 2005; Bailey et al., 2018), they represent a dramatic simplification of the larger biological context in which the studied biochemical reaction takes place. Here, plant related metabolization or uptake limitations of the compound due to the plant cuticula, cell walls or cell membrane are largely ignored. These restrictions are avoided when leaf cuttings or whole plants are used and compounds are tested at *in vivo* conditions (Haines et al., 2005; Motti et al., 2007). Compared to whole plant toxicity assays, oxygen evolution measurements on leaf cuttings are relatively easy and straightforward to perform using Clark-type electrodes with buffer-filled reaction chambers (Clark, 1956; Delieu and Walker, 1972). In this set-up the cuticular barrier is bypassed as water-soluble compounds are able to freely diffuse into the cells and within the symplast by the plasmodesmata exposed on the cut surfaces. Nevertheless, plant related metabolization of the compound may be still observable in these studies as most of the plant tissue is still largely intact. However, ensuring homogeneous illumination of all leaf cuttings for all repetitions and across different studies still remains challenging in this set-up. Stirring of the buffer solution naturally leads to unpredictable and random movement of the leaf slices, leaving no possibility to ensure that individual slices do not shade each other from the light source. Aside from these purely mechanical issues, earlier studies questioned the biological availability of dissolved CO_2_ (Jones and Osmond, 1973). The apparent K_m_ determined for CO_2_ was larger than observed in gas phase experiments (Jones and Slatyer, 1972) or on isolated chloroplasts (Jensen and Bassham, 1966). Jones and Slatyer (1972) pointed out that this effect is largely dependent on the experimental procedure and plant species used to prepare the leaf slices and is likely to be caused by diffusion problems of CO_2_ from the buffer to the photosynthetically active cells. This dependence on experimental setup and biological system used for the experiments severely hampers the interpretation of comparative studies (Jones and Osmond, 1973).

On the other hand, whole plant toxicity assessments are rather time-consuming — ranging from days to weeks for each replicate — to set up and to perform (Lein et al., 2004; Burgos, 2015). The outcome strongly depends on general growth conditions which can be difficult to control and/or other parameters that are not immediately discernible to the experimenter. In whole plant assays, inhibitor uptake across the cuticular barrier into the apoplast is usually the success-limiting factor (Tice, 2001). Hence, whole plant experiments are commonly considered in later stages of the development of new herbicides. Today, a simple, scalable assay albeit as close as possible to *in planta* conditions is still missing. While leaf cuttings provide a more complex biological system with intact photosynthetic apparatus, technical (e.g. uniform illumination of the sample) as well as biological (free diffusion of compounds) limits confine their application. Here, we present a novel *in vivo* test system using intact plant cell suspensions that builds up on previous studies using leaf cuttings (Haines et al., 2005; Motti et al., 2007; Minges and Groth, 2017).

Toxicity tests using heterotrophic plant cell cultures are routinely used (Olofsdotter et al., 1994) and do not suffer from the same disadvantages as whole plant or leaf cutting assays. However they are not applicable to the analysis of photosynthesis inhibitors. Similarly, isolated chloroplasts have been used to study photosynthesis and photosynthetic oxygen evolution (POE) (Jensen and Bassham, 1966). However, cellular environment such as membranes and detoxification mechanisms is missing. In the context of C_4_ photosynthesis, its complex apparatus of CO_2_ concentration mechanisms is absent and as such isolated chloroplasts are of limited use for the identification of C_4_-specific inhibitors of photosynthesis.

*Bienertia sinuspersici* is a succulent that belongs to the Suaedoideae subfamily of Amaranthaceae and features SCC_4_ within dimorphic chloroplasts (Freitag and Stichler, 2002; Akhani et al., 2005). Furthermore, *B. sinuspersici* chlorenchyma cells occur in loosely packed layers which simplifies the preparation of a functional cell suspension (Akhani et al., 2005; Offermann et al., 2011). Previous studies suggest that the difference between SCC_4_ and Kranz-type C_4_ is mainly of morphological, but not of biochemical nature (Lara et al., 2006; Lara et al., 2008; Park et al., 2010; Offermann et al., 2011; Offermann et al., 2015). Hence, most findings regarding biochemical mechanisms and key enzymes of C_4_ photosynthesis should generally be transferable between SCC_4_ and Kranz-type C_4_ plants.

## Materials and Methods

### Plant Material

*B. sinuspersici* plants were grown in growth chambers (CLF Plant Climatics, DE) as previously described (Leisner et al., 2010; Offermann et al., 2011) with day/night cycle (30°C/18°C, 14 h/10 h) and a photosynthetic flux density of 500 µmol quanta m^−2^ s^−1^. Plants were watered twice a week. Liquid fertilizer (0.2 % (v/v), NPK 12-4-6, Wuxal Top N, Manna, DE), and 100 mM NaCl were provided once per week while watering the plants. *Zea mays* was grown in the greenhouse under natural light conditions.

### Preparation of Chlorenchyma Cell Suspension

Medium-sized and well-illuminated mature leaves were harvested from the upper third of young *B. sinuspersici* plants. Leaves were gently squeezed with mortar and pestle in cold extraction buffer (750 mM betaine, 2.5 mM MgCl_2_, 2.5 mM NaH_2_PO_4_, 0.5 mM MnCl_2_, 25 mM HEPES, pH 7.5) for 5 to 10 min. A total volume of 5 ml buffer was used for the extraction of 50 leaves per preparation. The obtained cell suspension was centrifuged twice (100 × *g*, 4°C, 5 min). The supernatant of each step was discarded to segregate intact cells from cell debris and free chloroplasts. The green-colored sediment was re-suspended in the same volume of degassed extraction buffer used for the initial cell extraction step (Offermann et al., 2011). Chlorenchyma cell suspensions were kept in the dark on ice until usage in the O_2_ evolution assay. If necessary, cells were further diluted to yield an O_2_ evolution rate of approx. 10 nmol ml^−1^ min^−1^.

### Quality of Cell Suspensions

**Figure 1:**
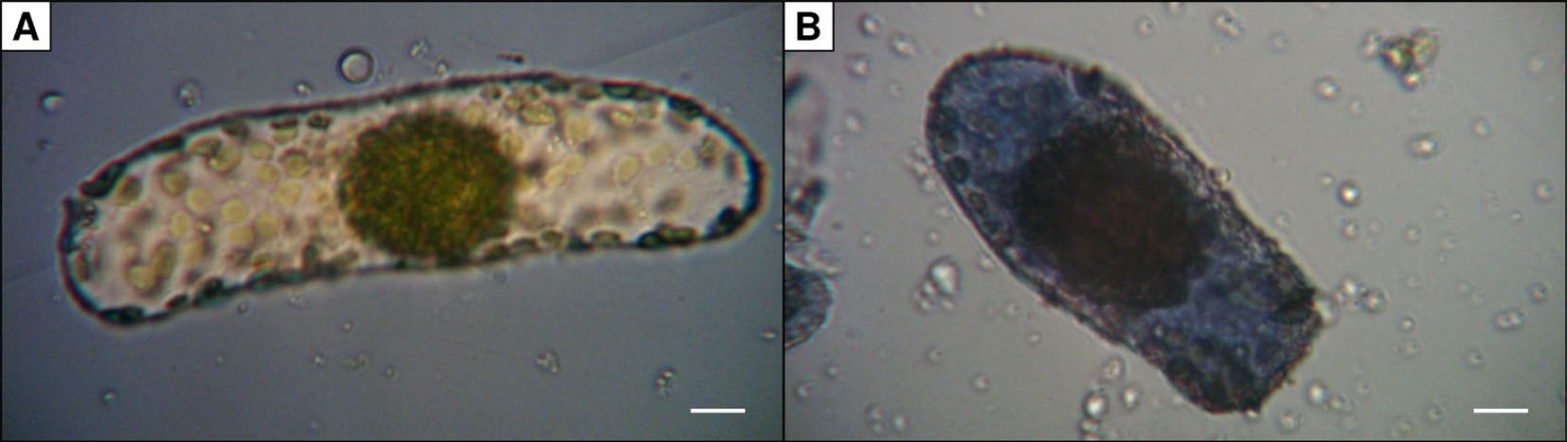
Trypan blue staining of isolated *Bienertia sinuspersici* chlorenchyma cells. Damaged cells **(B)** are stained due to diffusion of the dye into the cell whereas intact cells **(A)** remain unstained (scale bars = 10 µm).

Vital staining of *B. sinuspersici* cells was performed by mixing 45 µl of cell suspensions with 5 µl of 2% (w/v) trypan blue staining solution (Tennant, 1964). A fifth of the stained cell suspension was transferred to a Neubauer counting chamber. A light microscope (M11, Wild, Heerbrugg, CH) with attached digital camera (MikrOkular, Bresser, DE) was used for analysis and documentation (**Figure 1**). The ratio of intact (unstained) and damaged cells (stained) was calculated. In preparations used for measurements of oxygen evolution rate, 50% to 80% of cells were intact judged by trypan blue staining with a cell concentration about 100,000 to 450,000 cells ml^−1^.

### Measurements of Oxygen Evolution Rates

Measurements were performed using a Clark-type oxygen electrode (Chlorolab 2+ with light source LED1, Hansatech Instruments, Norfolk, UK) (Delieu and Walker, 1972). Pyruvate (1 mM) and NaHCO_3_ (1 mM) were added to 1 ml *B. sinuspersici* chlorenchyma cell suspension in extraction buffer to prevent substrate limitation. The sample was then incubated at 30°C for 5 min and added to the pre-heated (30°C) electrode chamber. Oxygen evolution in the dark was monitored until stability (dark rate). Two factors contribute to the dark rate: oxygen depletion by the electrode (electrode drift) and mitochondrial respiration. Subsequently the electrode chamber was illuminated at 1,000 µmol m^−2^ s^−1^ and light-dependent oxygen evolution (light rate) was recorded after signal stabilization. The POE which is indicative for the photosynthetic activity was calculated as the difference between light and dark rate.

Inhibition experiments were performed in the presence of known inhibitors of PEPC or PPDK. Here we used the chalcone okanin (Carbosynth, Compton, UK) — an allosteric inhibitor of PEPC activity — and bisindolylmaleimide IV (BIM4) (Cayman Chemicals, Ann Arbor, US) — an active-site inhibitor competing with ATP for the nucleotide binding site of PPDK.

Inhibitors were added to the electrode chamber after the system reached equilibrium. The rate was recorded (inhibited light rate), once a stable POE was reached again after addition of the compound. The relative inhibition was then calculated according to equation 1.

(1) $$\text{inhibition }[\%]=100-\frac{\text{POE}(\text{uninhibited)}}{\text{POE}(\text{inhibited})}\cdot 100$$

For okanin a substantial light-independent oxygen consumption was observed immediately after addition of the compound. However, this oxygen scavenging effect is well-known for other hydroxylated chalcones (Rathmell and Bendall, 1972; Haraguchi et al., 1998). To correct for this effect, the rate of oxygen consumption in the dark after addition of okanin was determined at every titration step and subtracted from the inhibited light rates.

Half-maximal (50%) inhibitory concentrations (IC_50_) were calculated by measuring the activity in the presence of different concentrations of each compound. Nine inhibitor concentrations in the range of 0 to 350 µM (BIM4) or 0 to 3,000 µM (okanin) were measured in triplicates as described above. A log-logistic dose response curve with three parameters (LL3) was globally fitted to the individual replicates using the R software collection v3.5.0 (R Core Team, 2018) and the R package drc v3.0-1 (Ritz et al., 2015).

### Comparison of Cell Suspension and Leaf Cutting Assays

Samples of *B. sinuspersici* cell suspensions and *Z. mays* leaves were taken on different days over the course of 6 h resulting in three measurement series for each type of sample. *B. sinuspersici* cell suspensions were prepared once for each measurement series as described previously and kept on ice. Samples of *Z. mays* originating from the same leaf and sized approx. 2 cm^2^ were harvested from the plant immediately before each measurement. The midrib was removed before samples were further processed into slices of approx. 1 mm thickness and placed in the measurement chamber filled with 1 ml of buffer containing 0.33 M sorbitol, 2.5 mM MgCl_2_, 2.5 mM NaH_2_PO_4_, 25 mM HEPES/KOH (pH 7.5), 50 μM MnCl_2_, and 2.5 mM dithiothreitol (Burnell and Hatch, 1988; Haines et al., 2005). Sodium bicarbonate and sodium pyruvate were added at 4 mM to initiate C_4_-driven photosynthesis. POE rates were monitored as stated before Leaf slices were recovered from the chamber and transferred to 1 ml of 80 % acetone (v/v) for chlorophyll extraction. Samples in acetone were kept in the dark at 4°C for 48 h. Absorption was measured using a DU800 spectrophotometer (Beckman Coulter, USA) at 645 and 663 nm and the total chlorophyll amount was calculated as stated in equation 2 (Arnon, 1949). Chlorophyll content was subsequently used for normalization of the POE rates expressed in nmol min^−1^ μg chl^−1^.

(2) $$\text{chl }[\mu \text{g }mL^{-1}]=(20.2\cdot OD_{645})+(8.02\cdot OD_{663})$$

Each measurement of a series was further normalized to the respective mean of the series. Data were processed with the R software collection v3.5.0 (R Core Team, 2018). Data were verified to be normally distributed using a Shapiro-Wilk’s test in combination with visual inspection of density and quantile-quantile plots. Variances of both sample types were statistically analyzed using an F-test with a significance level of α = 0.05.

### Multiple Sequence Alignment

Protein sequences of PEPC and PPDK from several C_4_ plants were selected on the basis of sequence quality and availability of PPDK and PEPC sequences for each species. They were aligned using MAFFT v7.407 in L-INS-i mode with default parameters (Katoh and Standley, 2013). Sequences used for the alignments are (UniProt identifiers if not stated otherwise): Bs (*B. sinuspersici*, PEPC: GenBank ABG20459.1, PPDK: GenBank GCEP01054245.1/GCEP01053558.1), Ft (*Flaveria trinervia*, PEPC: P30694, PPDK: P22221), So (*Saccharum officinarum*, PEPC: Q9FS96, PPDK: Q9SNY6), Sb (*Sorghum bicolor*, PEPC: P15804, PPDK: Q84N32), Zm (*Z. mays*, PEPC: P04711, PPDK: P11155), Ah (*Amaranthus hypochondriacus*, PEPC: E2JE39, PPDK: F8UU33), Ec (*E. crus-galli*, PEPC: Q52NW0, PPDK: Q4JIY1), and Si (*Setaria italica*, PEPC: Q8S2Z8, PPDK: A0A290Y0Z5). Figures of multiple sequence alignment (MSA) were prepared using TeXshade (Beitz, 2000).

### Homology Modeling and Ligand Docking

Ten homology models each of *B. sinuspersici* PEPC and PPDK were generated using MODELLER v9.21 (Webb and Sali, 2016). The models were ranked based on their normalized DOPE score (zDOPE) (Shen and Sali, 2006; Pieper et al., 2010) and the top-ranked model was used after visual inspection for docking of inhibitors into the proposed binding sites. Templates used for modeling of BsPEPC include PDB IDs 3ZGE, 3ZGB, 4BXH, 4BXC, 1JQO, 1QB4, and 5VYJ. Template selection for modeling of BsPPDK was limited to PDB ID 5JVL due to a high conformational diversity between the deposited structures. Ligands bound to the inhibitor and/or substrate binding sites were left in place during modeling to preserve side chain conformations and interactions within the binding sites.

Okanin and BIM4 were docked into the malate/aspartate binding site of BsPEPC or the nucleotide binding pocket of BsPPDK respectively. Charges and protonation state of the C_4_ enzymes were added by tools from the UCSF Chimera software suite (Pettersen et al., 2004). A total of 200 initial conformers of each compound, okanin and BIM4, were generated using RDKit (RDKit, 2019). Conformers with RMSDs below 0.1 Å were removed and the remaining set was used for docking using the AutoDock vina-derived software smina and the vinardo scoring function (Trott and Olson,2009; Koes et al., 2013). Side chains within a distance of 3 Å of the compound present in the original models (aspartate for BsPEPC and 2′-Br-dAppNHp for BsPPDK) were treated as being flexible during docking. The bounding box was sized 30 Å in each dimension and centered at the C_α_ atom of bound aspartate (BsPEPC) or the 4′-carbon atom of 2′-Br-dAppNHp (BsPPDK), respectively. Energy minimized docking poses were subsequently ranked according to their predicted binding affinities. Figures of molecular models were prepared using PyMOL (Schrödinger, 2017).

## Results and Discussion

### Photosynthetic Oxygen Production in SCC_4_ Cells Responds to Known C_4_ Inhibitors

The photosynthetic activity of *B. sinuspersici* chlorenchyma cell suspensions was determined by a Clark type oxygen electrode as described above. The yields of typical preparations were in the range of 1,000 to 7,200 intact cells per leaf judged by trypan blue staining. This results in a count of 50,000 to 360,000 intact cells used per measurement. During the initial incubation phase in the dark, a clear decrease in oxygen concentration due to electrode drift and mitochondrial respiration was observed. Upon illumination oxygen concentration in the electrode chamber increased due to photosynthetic activity of the cell suspension, allowing the analysis of C_4_ photosynthesis of *B. sinuspersici* cells *in vivo*. Optimal ranges of common control parameters such as pH, osmolarity, and temperature were identified allowing measurements of cell suspensions with stable oxygen rates for several hours. Thereby effects of known inhibitors of key enzymes of the C_4_ photosynthetic cycle can be tested at cellular conditions.

The two inhibitors analyzed in this study target different key enzymes of C_4_ photosynthesis. While the non-competitive inhibitor okanin binds to an allosteric regulatory site in PEPC, the competitive inhibitor BIM4 targets the nucleotide binding site of PPDK. Sequence alignments and phylogenetic analysis of both target enzymes across different C_4_ species including *B. sinuspersici* suggest that key motifs such as regulatory and active side residues are highly conserved. Hence, results obtained on *B. sinuspersici* cell suspension system presented here, may be transferred and generalized to other C_4_ species including typical weed species. Notwithstanding the observed conservation on sequence level, subtle differences across species — in particular for the allosteric feedback inhibition site of PEPC — have to be kept in mind.

**Figure 2:**
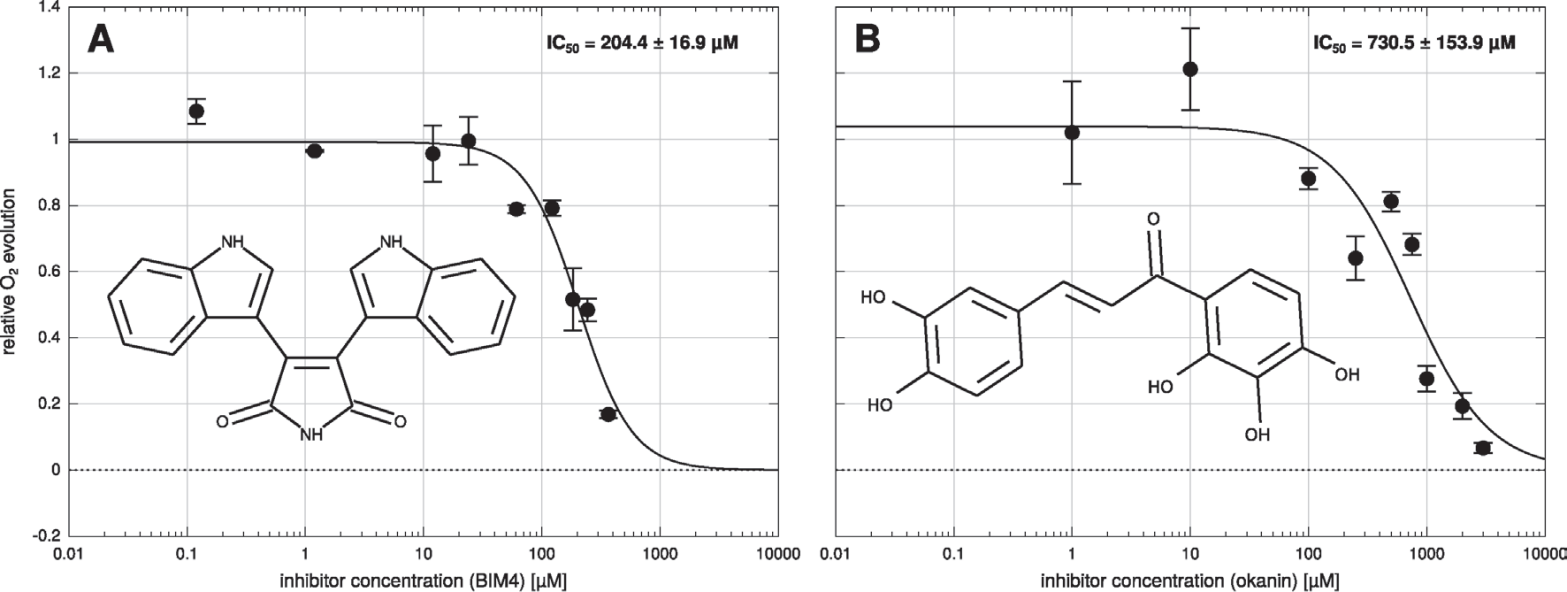
Dose response curve of *Bienertia sinuspersici* cell suspension. Bisindolylmaleimide IV (BIM4) **(A)** or okanin **(B)** were added in nine different concentrations to the cell suspension and photosynthetic oxygen evolution was measured. IC_50_ values were calculated by a globally fitting a three parameter log-logistic dose response curve to the individual data points. Errors shown are standard errors of the mean (SEM).

Measurements on isolated *B. sinuspersici* chlorenchyma cell suspensions show that both inhibitors significantly decrease photosynthetic oxygen production. For both compounds, inhibition is concentration dependent. From the corresponding activity curves (**Figure 2**) IC_50_ of 204 ± 17 µM were obtained for BIM4 and 731 ± 154 µM for okanin. In contrast, IC_50_ concentrations in the upper nanomolar range have been reported for both inhibitors for the purified enzymes — precisely 0.8 ± 0.2 µM for BIM4 and 0.6 ± 0.1 µM for okanin (Nguyen et al., 2016; Minges et al., 2017). The IC_50_ concentrations observed under *in vitro* and *in vivo* conditions differ by almost three orders of magnitude which is probably owed to uptake limitations across the cell membrane and/or plant related metabolization of the active compounds. For okanin the higher IC_50_ observed in the *in vivo* studies on plant cell suspension might also issue from sequence variation in a key residue of the allosteric binding site. In *B. sinuspersici* this residue at position 884 (*Flaveria* numbering) corresponds to arginine, whereas the *Flaveria* C_4_ model used in the *in vitro* studies (Paulus et al., 2013a; Paulus et al., 2014; Nguyen et al., 2016) carries a glycine at this position allowing unrestricted access to the allosteric binding site.

**Figure 3:**
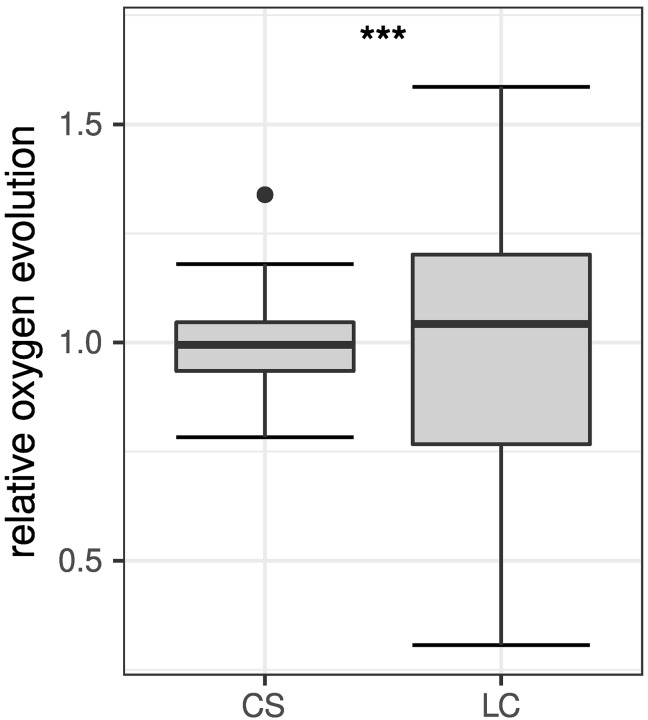
Comparison of relative POE obtained from cell suspension (CS, n = 30) and leaf cutting (LC, n = 21) assays. Data was normalized to the respective mean of each measurement series for better comparability and are shown as boxplots (Tukey 1977). The box represents the interquartile range (IQR) while the median is depicted by a solid line. Whiskers include data within 1.5 × IQR. Data points beyond this range are shown individually depicted by a filled circle (•). The difference between variances of both datasets is statistically highly significant (***p ≤ 0.001) according to F-test results (F = 0.12332, p = 6.988 · 10^−7^).

To explore whether the cell suspension assay is able to yield more accurate data compared to leaf cutting assays, measurements of POE were repeated on different days over the course of 6 h as described in as described in Material and Methods For better comparability, POE rates obtained from both methods were normalized to the respective mean of each measurement series (**Figure 3**). Analysis of variances for both methods clearly shows a narrower distribution of data for the cell suspension assay [standard deviation (sd) = 0.12] than for the leaf slice assay (sd = 0.33). Statistical analysis using an F-test suggest that the observed difference in variances between both methods is highly significant (F = 0.12332, p = 6.998 · 10^−7^).

### Structural Comparison of PEPC and PPDK Inhibitor Binding Sites Among Different C_4_ Species

**Figure 4:**
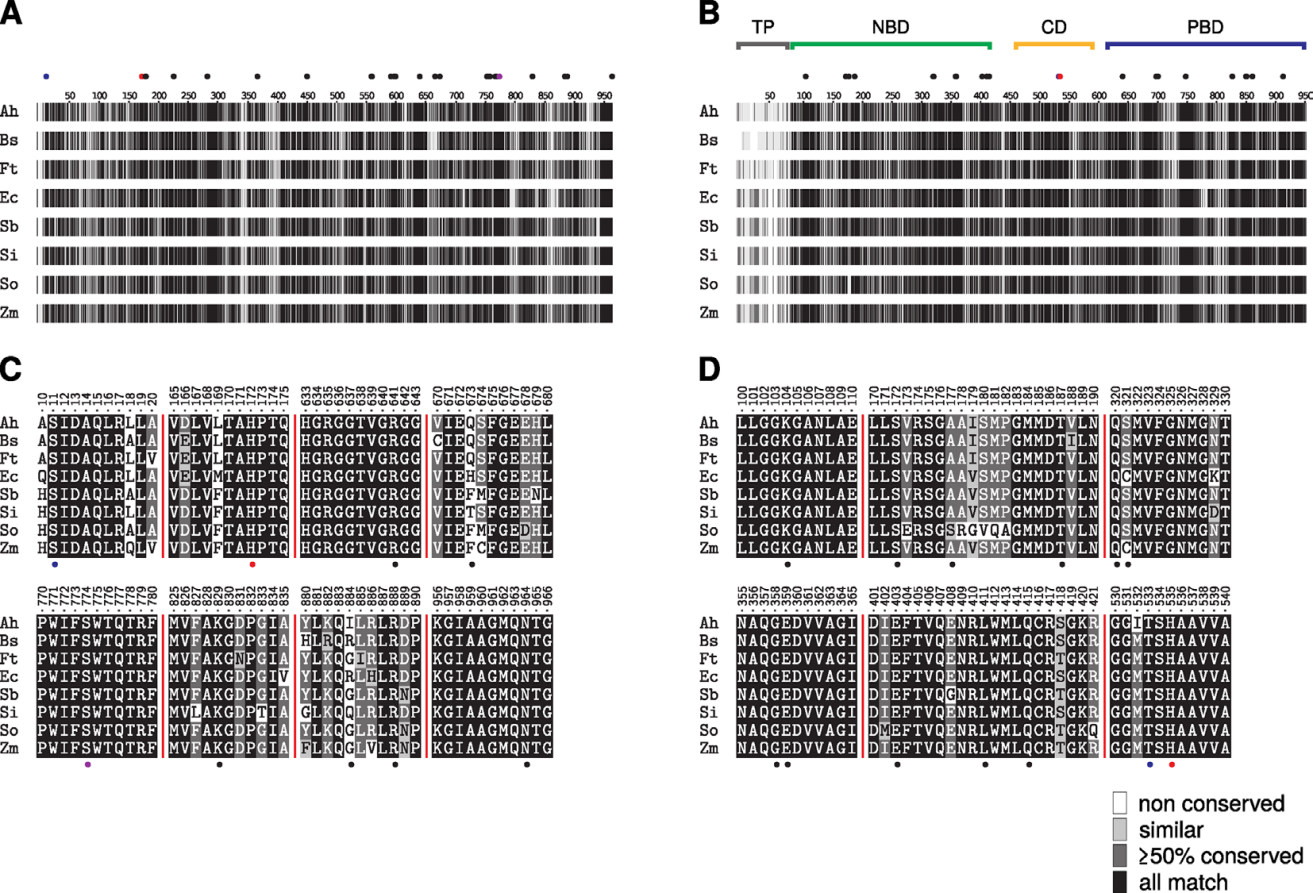
Multiple sequence alignment of phosphoenolpyruvate carboxylase (PEPC) **(A, C)** and pyruvate phosphate dikinase (PPDK) **(B, D)** sequences of representative C_4_ plants. The complete alignment is visualized as a condensed fingerprint in **(A** and **B)**. Sequence similarity according to BLOSUM62 substitution matrix is depicted as grayscale gradient, ranging from black (full match) to white (not conserved). Sequence numbering corresponds to *Flaveria trinervia* (Ft) PEPC and PPDK. The substrate binding domains of PPDK (nucleotide: NBD, PEP/pyruvate: PBD), its catalytic central domain (CD). and the non-conserved chloroplastic transit peptide (TP) are marked as bars above the fingerprint **(B)**. Cut-outs of regions important for inhibitor binding or catalytic activity are shown in panels **C** (PEPC) and **D** (PPDK) respectively. Residues involved in substrate or effector binding are depicted as solid black dots while catalytic residues are colored in red. Residues marked with blue dots are of regulatory function. S774 of PEPC **(A, C)** depicted in purple has been described as a specific marker for C_4_-type PEPCs (Svensson et al., 2003). All highlighted positions are located in highly conserved regions with the exception of positions 673 and 884 in PEPC **(C)**. Full-length sequence alignment is available from the **Supplementary Material**.

A critical prerequisite to generalize and transfer findings obtained with the *B. sinuspersici* cell suspension assay presented in this study is that key enzymes and mechanisms in the SCC_4_ plant function in a similar manner as in conventional Kranz-type C_4_ plants. To identify possibly meaningful differences on sequence level, a MSA of the two key enzymes of C_4_ photosynthesis — PEPC and PPDK — was computed for a range of C_4_ species (**Figure 4**). The obtained MSA (see **Supplementary Material**) confirms that primary sequences of both C_4_ enzymes are highly conserved between the analyzed C_4_ plants (**Figures 4A, B**) in particular their substrate and effector binding sites or their catalytic core (**Figures 4C, D**). For PPDK, substrate binding can be allocated to two distinct domains: the N-terminal nucleotide-binding domain (NBD) and the C-terminal PEP/pyruvate-binding domain (**Figure 4B**). Both are separated by a flexible central domain which houses a regulatory threonine and a catalytic histidine residue (Carroll et al., 1994; Herzberg et al.,1996). Previous studies indicated that the PPDK inhibitor BIM4 binds to the NBD as an ATP competitive inhibitor (Minges and Groth, 2017). Consequently, the analysis of the PPDK MSA was focused on regions and residues directly involved in nucleotide binding (**Figure 4D**).

While residues are fully conserved at the PPDK nucleotide binding site and the PEPC C-terminal tetrapeptide needed for optimal catalytic activity (Dong et al., 1999; Kai et al., 2003) the MSA indicates obvious variance at positions 673 and 884 (*F. trinervia* numbering) for PEPC. This region is part of the PEPC allosteric aspartate/malate inhibitor site and is likewise involved in binding the inhibitor okanin (Nguyen et al., 2016). Although, previous studies disclosed that residues at position 673 contribute to the binding of PEPC feedback inhibitors aspartate and malate (Paulus et al., 2013b), no particular role of those residues for C_4_-typical kinetics has been demonstrated yet. Notably position 884 has been demonstrated in previous *in vitro* studies of critical importance to modulate the binding affinity of C_4_ acids malate and aspartate (Paulus et al., 2013a; Paulus et al., 2013b). C_3_-type PEPCs have a conserved R884 at this position that further stabilizes aspartate/malate binding at the inhibitor site. Crystallographic studies in combination with mutational analysis and activity studies have shown that substitution of R884 by glycine significantly decreased the binding affinity of PEPC for aspartate and malate and thereby the sensitivity of PEPC toward feedback inhibition (Paulus et al., 2013a; Paulus et al., 2013b). Many C_4_-type PEPCs carry glycine at position 884, but also serine, glutamine, and glutamate are found for C_4_-type PEPCs at this position which also provide decreased feedback inhibition (Paulus et al., 2013a). Previous studies suggest that a bulky amino acid such as arginine at position 884 interferes with the binding of the C_4_-specific inhibitor okanin (Paulus et al., 2014; Nguyen et al., 2016). Remarkably enough, the C_3_-typical arginine is also found at position 884 in a range of C_4_ PEPCs particularly in the C_4_ grass lineages as indicated by the MSA (**Figure 4C**). Though, it is not clear yet whether other mutations at the allosteric inhibitor site counteract the higher binding affinity for C_4_ acids aspartate/malate caused by R884 for these enzymes. Moreover it has been hypothesized that C_4_ characteristics may rather not be determined by the presence of C_4_-typical amino acids, but by the absence of non-C_4_ ones at certain positions at the active or allosteric sites (Christin et al., 2007). Along the same lines, in recent studies, Rosnow et al. (2014; 2015) compared PEPC sequences in the C_4_ Suaedoideae clade and did not notice any convergence in amino acid substitutions at the allosteric feedback inhibitor binding site. However, they observed a convergence of kinetic parameters such as a lower PEP affinity of C_4_ PEPCs compared to their C_3_ isoforms which presumably allows for the accumulation of higher PEP concentrations in the C_4_ Suaedoideae (Bläsing et al., 2000).

**Figure 5:**
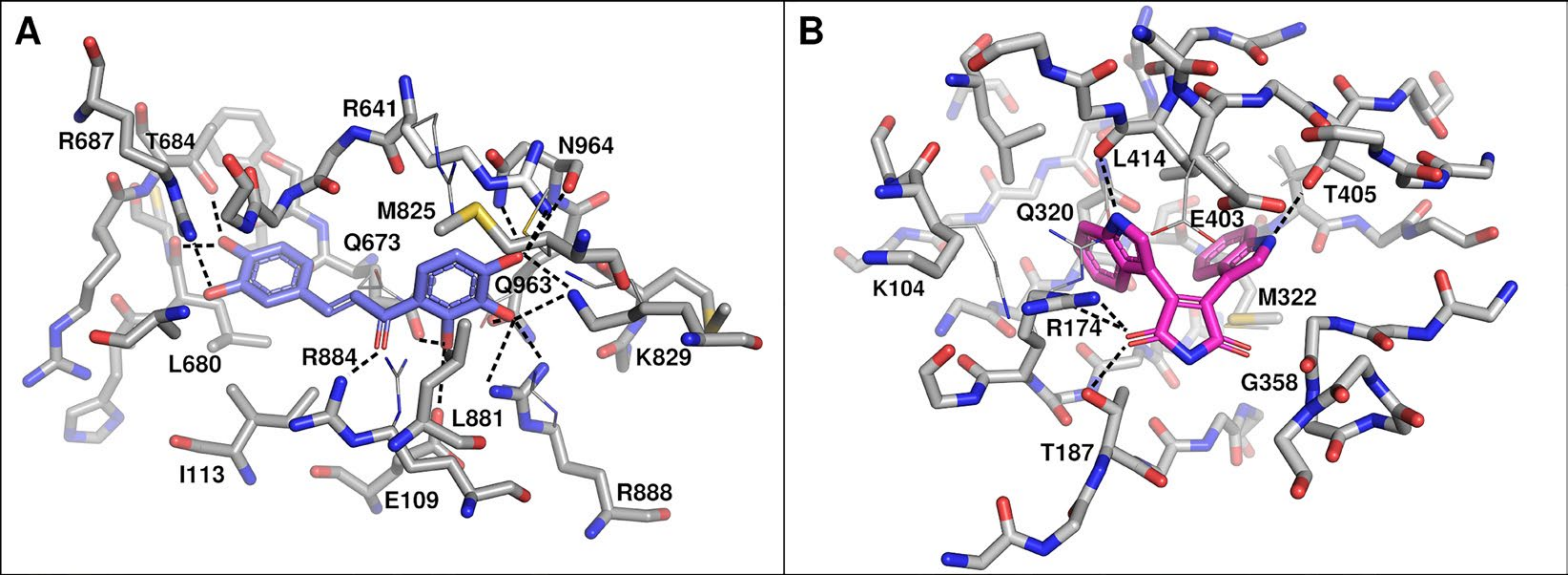
Docked binding poses of okanin **(A)** and bisindolylmaleimide IV (BIM4) **(B)**. Residues within 3 Å of the compound are labeled (*Flaveria trinervia* nomenclature) and polar interactions are shown as dashed lines. Side chain conformations after docking are shown as sticks. For side chains that have been displaced during flexible docking, the original conformation is shown in line representation. In case of okanin and BsPEPC **(A)**, residues M825, R641, and R884 assumed a substantially different side chain conformation. However, only the original orientation of R884 would have been of steric hindrance. In case of BIM4 and BsPPDK, residues K104, R174, and E403 have been displaced with the original side chain orientation of the latter one being of steric hindrance to BIM4 binding.

To verify the possibility of okanin binding to BsPEPC despite containing an arginine at position 884 and to verify the proposed binding mode of BIM_4_ within the nucleotide binding site of BsPPDK, homology models of both enzymes were calculated. Combined with virtual docking of okanin and BIM4, these models substantiated the structural similarity of the two key enzymes of C_4_ photosynthesis in *B. sinuspersici* compared to those of other C_4_ plants. In its best-scored docking pose, okanin forms polar contacts to the side chains of E109, R641, T684, R687, K829, R884, and N964 (**Figure 5A**). In this context, the involvement of R884 is unexpected as it is thought to be the key switch in mediating sensitivity of C_4_ PEPCs toward their feedback inhibitors aspartate and malate as well as for the selective binding of okanin to C_4_ PEPCs (Paulus et al., 2013a; Paulus et al., 2013b; Nguyen et al., 2016). In its orientation resolved in the crystal structures of model C_4_ PEPC from *F. trinervia*, R884 indeed provides substantial steric hindrance and thus may shield the site from okanin binding (Paulus et al., 2013b; Schlieper et al., 2014). Interestingly though, our recent docking studies on the BsPEPC homology model suggest that the R884 side chain is displaced upon okanin binding. The observed side chain flexibility might account for the inhibitory effects of okanin on PEPC observed in the *in vitro* (Nguyen et al., 2016) and *in vivo* experiments presented in this study.

For BsPPDK, docking results of BIM4 are largely congruent with those previously obtained for *F. trinervia* PPDK (Minges and Groth, 2017) with predicted binding affinities up to −50.208 kJ mol^−1^ (−12.0 kcal mol^−1^). The docking suggests that BIM4 is bound *via* polar side chain contacts to R174, T187, and T405 (**Figure 5B**). Similar to the situation observed for okanin, individual side chains (R174 and E403) are shifted upon inhibitor binding in comparison to their initial conformation in the homology models.

## Conclusion

Suspensions of intact *B. sinuspersici* chlorenchyma cells provide an easy to use, but yet powerful system to study the selective effect of small molecule inhibitors on C_4_ photosynthesis in a plant context. Compared to measurements on leaf cuttings the *Bienertia* system is easier in handling, higher in performance and has better reproducibility. The homogeneous *Bienertia* cell suspensions used in this set-up ensure an uniform distribution of compounds and constant illumination across the sample. Moreover, inhibitor uptake from the apoplast, but also the cell membrane are still valid in this system as isolated cells have intact cell membranes. The cell suspension assay itself is easily scalable. The assay volume per measurement is limited only by the type of electrode chamber. Setups with smaller or larger volumes are possible as well as parallel measurements using multiple Clark-type electrodes or fluorescence-based assays in a 96 well scale (Deshpande et al., 2004; Arain et al., 2005). Altogether, the *in vivo* approach presented in this study expands our methodical repertoire to further study the molecular processes of C_4_ photosynthesis and to foster the development of C_4_-specific inhibitors.

## Data Availability Statement

The datasets generated and analyzed for this study can be found in the Zenodo repository doi: 10.5281/zenodo.2635517.

## Author Contributions

GG designed research. SO and AM contributed to experimental design. DJ performed cell preparation and oxygen measurements. DJ and AM performed data analysis. AM performed bioinformatical analysis. AM, DJ and GG wrote the manuscript. All authors read and edited the manuscript prior to publication.

## Funding

Funded in part by the Deutsche Forschungsgemeinschaft (DFG, German Research Foundation) under Germany´s Excellence Strategy — EXC 2048/1 — Project ID: 390686111. Work in the S.O. lab was funded by the “Cooperative Research Program for Agriculture Science & Technology Development (Project No. 0109532016)”. Rural Development Administration, Republic of Korea.

## Conflict of Interest

The authors declare that the research was conducted in the absence of any commercial or financial relationships that could be construed as a potential conflict of interest.
