# Supplementary material for: Efficient In Vivo Screening Method for the Identification of C4 Photosynthesis Inhibitors Based on Cell Suspensions of the Single-Cell C4 Plant Bienertia sinuspersici
Source: Front Plant Sci. 2019 Oct 30;10:1350. doi: 10.3389/fpls.2019.01350 (PMC6831552; doi:10.3389/fpls.2019.01350)
Supplement: Supplementary file 1 [file DataSheet_1.pdf]

frontiers

Supplementary Material: Efficient *in vivo* screening method for the identification of C_4 photosynthesis inhibitors based on cell suspensions of the single-cell C_4 plant *Bienertia sinuspersici*

1 SUPPLEMENTARY DATA

Sequences used for the alignments are (UniProt identifiers if not stated otherwise): Bs (*Bienertia sinuspersici*, PEPC: GenBank ABG20459.1, PPDK: GenBank GCEP01054245.1/GCEP01053558.1), Ft (*Flaveria trinervia*, PEPC: P30694, PPDK: P22221), So (*Saccharum officinarum*, PEPC: Q9FS96, PPDK: Q9SNY6), Sb (*Sorghum bicolor*, PEPC: P15804, PPDK: Q84N32), Zm (*Zea mays*, PEPC: P04711, PPDK: P11155), Ah (*Amaranthus hypochondriacus*, PEPC: E2JE39, PPDK: F8UU33), Ec (*Echinochloa crus-galli*, PEPC: Q52NW0, PPDK: Q4JIY1), Si (*Setaria italica*, PEPC: Q8S2Z8, PPDK: A0A290Y0Z5).

1.1 Multiple Sequence Alignment of PEPC

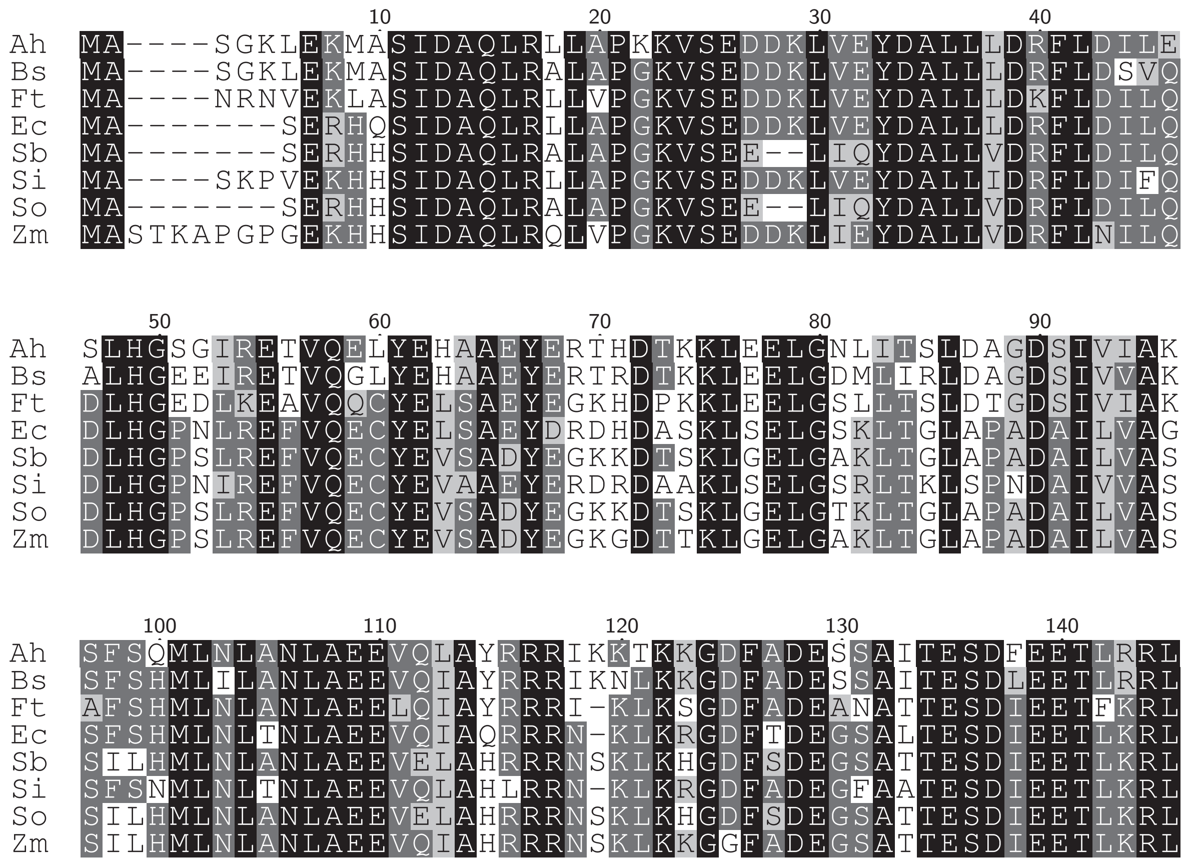

```
          150       160       170       180       190
Ah  V-DLKESPEEIFATLKNQTVDLVLTAHPTQSVRRSLLQKHGRIRDCLSQL
Bs  VVDLKKSPEEIFETLKNQTVELVLTAHPTQSVRRSLLQKHGRIRDCLTQL
Ft  VHKLNKSPEEVFDALKNQTVELVLTAHPTQSVRRSLLQKHGRIRNCLAQL
Ec  VTDLGKTKEEVFDALKNQIVELVMTAHPTQSVRRSLLQKYSRIRNCLTEL
Sb  VS-LGKTPAEVFEALKNQSVDLVFTAHPTQSARRSLLQKNARIRNCLTQL
Si  VTELGKSKEEVFDALKNQTVDLVFTAHPTQSVRRSLLQKHARIRNCLTQL
So  VS-LGKTPEEVFEALKNQSVDLVFTAHPTQSARRSLLQKNARIRNCLTQL
Zm  VSEVGKSPEEVFEALKNQTVDLVFTAHPTQSARRSLLQKNARIRNCLTQL

          200       210       220       230       240
Ah  YAKDITPDDKQELDEALQREIQAAFRTDEIRRVQPTPQDEMRMGMSYFHE
Bs  YAKDITPDDKQELDEALQREIQAAFRTDEIRRMQPTPQDEMRAGMSYFHE
Ft  YAKDITPDDKQELDEALHREIQAAFRTDEIRRTPPTPQDEMRAGMSYFHE
Ec  YAKDISADDKKELDEALQREIQAAFRTDEIRRAQPTPQDEMRYGMSYFHE
Sb  SAKDVTVEDKKELDEALHREIQAAFRTDEIRRAQPTPQDEMRYGMSYIHE
Si  YAKDNTEDDKQELDEALQREIQACFRTDEIRRAQPTPQDEMRYGMSYFHE
So  STKDVTVEDKKELDEALQREIQAAFRTDEIRRAQPTPQDEMRYGMSYIHE
Zm  NAKDITDDDKQELDEALQREIQAAFRTDEIRRAQPTPQAEMRYGMSYIHE

          250       260       270       280       290
Ah  TIWKGVPKFLRRVDTALKNIGINERVPYNVPLIQFSSWMGGDRDGNPRVT
Bs  TIWKGVPKFLRRLDTALKNIGINERVPYNAPLIQFSSSMGGDRDGNPRVT
Ft  TIWKGVPKFLRRVDTALKNIGINERFPYNAPLIQFSSWMGGDRDGNPRVT
Ec  TIWKGVPKFLRRVDTALKNIGIDERLPYNVPLIKFSSWMGGDRDGNPRVT
Sb  TVWNGVPKFLRRVDTALKNIGINERLPYDVPLIKFCSWMGGDRDGNPRVT
Si  TIWKGVPKFLRRVDTALKNIGIDERLPYNAPLIQFSSWMGGDRDGNPRVT
So  TVWKGVPKFLRRVDTALKNIGINQRLPYNVPLIKFCSWMGGDRDGNPRVT
Zm  TVWKGVPKFLRRVDTALKNIGINERLPYNVSLIRFSSWMGGDRDGNPRVT

          300       310       320       330       340
Ah  PEVTRDVVLLARMMAANMYFTQITDLMFELSMWRCNDEVRARAQELRSQ-
Bs  PEVTRDVVLLARMMAANMYFSQIQDLMFELSMWRCNDELNARAHEIHKL-
Ft  PEVTRDVCLLARMMTSNMYFSQIEDLMIEMSMWRCNSELRVRAEELYRT-
Ec  PEVTRDVCLLARMMAANLYFSQIEDLMFELSMWRCNDELRARADELLDSA
Sb  PEVTRDVCLLSRMMAANLYINQVEDLMFELSMWRCNDELRARAEEVQSTP
Si  PEVTRDVCLLARMMASNLYFSRIEELMFELSMWRCNDELRARAEELHAS-
So  PEVTRDVCLLSRMMAANLYIDQVEDLMFELSMWRCNDELRARAEEVQSTP
Zm  PEVTRDVCLLARMMAANLYIDQIEELMFELSMWRCNDELRVRAEELHSSS

            350       360       370       380       390
Ah  -SKSDAKHYIEFWKQIPLSEPYRVILGDVRDKLYNTREHAHQLLANGASD
Bs  -SKSDAKHYIEFWKPIPPSEPYRVVLADVRDKLYHTREHARQLLSNGTSD
Ft  -ARKDVKHYIEFWKRIPPNQPYRVILGDVRDKLYNTRERSRHLLVDGKSD
Ec  SKKTANKHYIEFWRGIPPNEPYRVVLGYVRDKLYNTRERSRHLLTSGFSE
Sb  ASKKVTKYYIEFWKQIPPNEPYRVILGAVRDKLYNTRERARHLLATGFSE
Si  -SQRISKHYIEFWRQLPANEPYRVVLGYVRDELYSTRERSRHLLTSGFSD
So  ASKKVTKYYIEFWKQIPPNEPYRVILGAVRDKLYNTRERARHLLATGFSE
Zm  GS-KVTKYYIEFWKQIPPNEPYRVILGHVRDKLYNTRERARHLLASGVSE

            400       410       420       430       440
Ah  VPEESTFTHIDQFLEPLELCYKSLCASGDRPIADGSLLDFMRQVSTFGLS
Bs  VPLESTFTHVDQFLEPLELCYKSLCDCGDRPIADGSLLDFMRQVSTFGLC
Ft  IPDEAVYTNVEQLLEPLELCYRSLCDCGDHVIADGSLLDFLRQVSTFGLS
Ec  ITEDLVFTNIEEFLEPLELCYKSLCECGDKIVADGSLLDFLRQVSTFGLS
Sb  ISEDAVFTKLEEFLEPLELCYKSLCECGDKAIADGSLLDLLRQVFTFGLS
Si  IPEDSAFKNVEEFLEPLELCYKSLCDCGDKTIADGSLLDFMRQVSTFGLS
So  ISVDSVFTNIEEFLEPLELCYKSLCDCGDKAIADGSLLDLLRQVFTFGLS
Zm  ISAESSFTSIEEFLEPLELCYKSLCDCGDKAIADGSLLDLLRQVFTFGLS
```

```
          450        460        470        480        490
Ah  LVKLDIRQESDRHTEVMDAITTHLGIGSYRSWSEEKRQEWLLSELRGKRP
Bs  LVKLDIRQESERHTDVMDAITKHLGVGSYREWSEEKRQEWLLSELRGTRP
Ft  LVKLDIRQESDRHTEVLDAITQHLGIGSYREWSEEKRQEWLLAELSGKRP
Ec  FVKLDIRQESERHTDAIDAITTHLGIGSYKSWPEEKRQEWLLSELQGKRP
Sb  LVKLDIRQESERQTDVIDAITTHLGIGSYRSWPEDKRMEWLVSELKGKRP
Si  MVKLDIRQESERHTDVIDAITTHLGIGSYREWSEEKRQEWLLSELRGKRP
So  LVKLDIRQESERHTDVIDAITTYLGIGSYRSWPEDKRMEWLVSELKGKRP
Zm  LVKLDIRQESERHTDVIDAITTHLGIGSYREWPEDKRQEWLLSELRGKRP
```

```
          500        510        520        530        540
Ah  LFGSDLPMSYEVADAIGTFRVLAELPNDSFGAYIISMATAPSDVLAVELL
Bs  LFGSDLPKTEEIAAVLDTFHVISELPSDGFGAYIISMATAPSDVLAVELL
Ft  LIGPDLPKTEEVKDCLDTFKVLAELPSDCFGAYIISMATSTSDVLAVELL
Ec  LLTPDPPTSDEVADVLGCFRVLAELPRDSFGPYIISMATAPSDVPAVELL
Sb  LLPPDLPMTEEIADVIGAMRVLAELPIDSFGPYIISMCTAPSDVLAVELL
Si  LLSKDMPQTEEIADVLGCFHVLAELPRDSFGPYIISMATAPSDVLAVELL
So  LLPPDLPMTEEIADVIGAMHVLAELPSDSFGPYIISMCTAPSDVLAVELL
Zm  LLPPDLPQTDEIADVIGAFHVLAELPPDSFGPYIISMATAPSDVLAVELL
```

```
          550        560        570        580        590
Ah  QRECGIKKPLRVVPLFEKLADLQSAAASMTRLFSIDWYKNRINGTQEVMI
Bs  QRECHIQNPLRVVPLFEKLADLENAPASITRLFSIDWYRNRINGKQEVMI
Ft  QREYHIKHPLRVVPLFEKLADLEAAPAAMTRLFSMDWYRNRIDGKQEVMI
Ec  QRECQIKDPLPVVPLFERLADLRAGPASVERLFSTEWYLKRINGKQQVMV
Sb  QRECGIRQTLPVVPLFERLADLQAAPASVEKLFSTDWYINHINGKQQVMV
Si  QRECHVKQPLPVVPLFEKLADLQSAPASIERLFSLDWYMNRIGGKQQVMV
So  QRECGIRQTLPVVPLFERLADLQAAPASVERLFSTDWYFDHIKGKQQVMV
Zm  QRECGVRQPLPVVPLFERLADLQSAPASVERLFSVDWYMDRIKGKQQVMV
```

```
          600        610        620        630        640
Ah  GYSDSGKDAGRLSAAWQLYKVQEQLIQVAKEYGVKLTMFHGRGGTVGRGG
Bs  GYSDSGKDAGRLSAAWQLYKAQEELIKIAKEFGVKLTMFHGRGGTVGRGG
Ft  GYSDSGKDAGRFSAAWQLYKTQEQIVKIAKEFGVKLVIFHGRGGTVGRGG
Ec  GYSDSGKDAGRLSAAWALYRAQEGMAEVAKKYGVQITFFHGRGGTVGRGG
Sb  GYSDSGKDAGRLSAAWQLYVAQEEMAKVAKKYGVKLTLFHGRGGTVGRGG
Si  GYSDSGKDAGRLSAAWHLYKAQEAMAKVAKRYGVKLTMFHGRGGTVGRGG
So  GYSDSGKDAGRLSAAWQLYVAQEEMAKVAKKYGVKLTLFHGRGGTVGRGG
Zm  GYSDSGKDAGRLSAAWQLYRAQEEMAQVAKRYGVKLTLFHGRGGTVGRGG
```

```
          650          660        670        680        690
Ah  GPTHLALLSQPP--DTIHGSLRVTIQGEVIEQSFGEEHLCFRTLERYTAA
Bs  GPTHLAILSQPPEHDSMD-HFVLPFKVKCIEQSFGEEHLCFRTLQRFTAA
Ft  GPTHLALLSQPP--DTINGSLRVTVQGEVIEQSFGEEHLCFRTLQRFCAA
Ec  GPTHLAILSQPP--NTINGSLRVTIQGEVIEHSFGEEHLCFRTLQRFTAA
Sb  GPTHLAILSQPP--DTINGSIRVTVQGEVIEFMFGEENLCFQSLQRFTAA
Si  GPTHLAILSQPP--DTINGSLRVTVQGEVIETSFGEEHLCFRTLQRFTAA
So  GPTHLAILSQPP--DTINGSIRVTVQGEVIEFMFGEDHLCFQSLQRFTAA
Zm  GPTHLAILSQPP--DTINGSIRVTVQGEVIEFCFGEEHLCFQTLQRFTAA
```

```
              700        710        720        730        740
Ah  TLEHGMHPPTSPKPEWRALMDEMAVITTKEYRSVVLQEPRFVEYFRSATP
Bs  TLEHGMHPPISPKPEWRTLLDEMAVAATKEYRSIVLKKPRFVEYFRLATP
Ft  TLEHGMNPPISPRPEWRELMDQMAVVATEEYRSVVFKEPRFVEYFRLATP
Ec  TLEHGMHPPASPKPEWRALMDEVTAVATEEYRSYVFKEPRFVEYFRSATP
Sb  TLEHGMHPPVSPKPEWRKLMEEMAVVATEEYRSVVVKEPRFVEYFRSATP
Si  TLEHGMHPPVSPKPEWRALMDEIAAVATDEYRSVVMREPRFVEYFRSATP
So  TLEHGMHPPVSPKPEWRKLMEEMAVVATEEYRSVVVKEPRFVEYFRSATP
Zm  TLEHGMHPPVSPKPEWRKLMDEMAVVATEEYRSVVVKEARFVEYFRSATP
```

```
        750       760       770       780       790
Ah  ELEYGRMNIGSRPAKRKPGGGIETLRAIPWIFSWTQTRFHLPVWLGCGAA
Bs  ELEYGRMNIGSRPSKRKPGGGIESLRAIPWIFSWTQTRFHLPGGLGCGAA
Ft  ELEFGRMNIGSRPSKRKPSGGIESLRAIPWIFSWTQTRFHLPVWLGFGAA
Ec  ETEYGRLNIGSRPAKRKPGGGTESLRAIPWIFSWTQTRFHLPVWLGFGAA
Sb  ETEYGKMNIGSRPAKRRPGGGITTLRAIPWIFSWTQTRFHLPVWLGVGAA
Si  ETEYGRLNIGSRPAKRKPKGGIESLRAIPWIFSWTQTRFHLPVWLGFGAA
So  ETEYGKMNIGSRPAKRKPGGGITTLRAIPWIFSWTQTRFHLPVWLGVGAA
Zm  ETEYGRMNIGSRPAKRRPGGGITTLRAIPWIFSWTQTRFHLPVWLGVGAA
```

```
        800       810       820       830       840
Ah  FKHVIEKDIK-NLAMLKDMYNQWSFFRVTIDLLEMVFAKGDPGIAALYDK
Bs  FKHVIEKDIK-NLAMLEQMYNEWPFFRVTIDLVEMVFAKGDPGIAALYDK
Ft  FKHAIQKDSK-NLQMLQEMYKTWPFFRVTIDLVEMVFAKGNPGIAALNDK
Ec  FNLRHQEGTSGNIQTLKEMYNEWPFFRVTLDLVEMVFAKGDPGIVGFYND
Sb  FKWAIDKDIK-NFQKLKEMYNEWPFFRVTLDLLEMVFAKGDPGIAGLYDE
Si  FEHAMKKDIK-NFRMLKEMYNEWPFFRVTLDLLEMVLAKGDPTIAGLYDQ
So  FKWAIDKDIK-NFQKLKEMYNEWPFFRVTLDLLEMVFAKGDPGIAGLYDL
Zm  FKFAIDKDVR-NFQVLKEMYNEWPFFRVTLDLLEMVFAKGDPGIAGLYDE
```

```
         850       860       870       880       890
Ah  LLVKDELKPFGENLRKSYLEAQKFLLEIAGHKDPLDADPYLKQILRLRDP
Bs  LLVSEELCPFGEQLRSDYEETKNFFFQIAGHKEILEGDPHLRQRLRLRDP
Ft  LLVSEDLRPFGESLRANYEETKNYLLKIAGHKDLLEGDPYLKQGIRLRDP
Ec  LLVADELKPFGEQLRKNYIDTCSFSCRLLGTRKSLEGDPYLKQRLHLRDP
Sb  LLVAEELKPFGKQLRDKYVETQQLLLQIAGHKDILEGDPYLKQGLRLRNP
Si  LLVADELKPFGEQLRNNYVETEKLILQVAGHKEILESDPGLKQQLRLRDP
So  LLVADDLKPFGKQLRDKYVETEKLLLQIAGHKDILEGDPYLKQGLRLRNP
Zm  LLVAEELKPFGKQLRDKYVETQQLLLQIAGHKDILEGDPFLKQGLVLRNP
```

```
         900       910       920       930       940
Ah  YTTTLNVFQVYTLKRIRDPSFHVTVRPHLSKEMDA--NSLAAELVKLNPT
Bs  YITTLNVCQAYTLKRIRDPNYHVTVRPHISKDYMDSTDKPAAELVKLNPS
Ft  YITTLNVCQAYTLKRIRDPNYHVTLRPHISKEYAAEPSKPADELIHLNPT
Ec  YITTLNVFQAYTLKRIRDPNFKVTLNPPLSNEFADE-NKPAG-LVKLNPA
Sb  YITTLNVFQAYTLKRIRDPSFKVTPQPPLSKEFADE-NKPAG-LVKLN-G
Si  YITILNVWQAYTLKRIRDPNFKVTPQPPLSKEFADE-NQPRG-IVKLNPA
So  YITTLNVLQAYTLKRIRDPCFKVTPQPPLSKEFADE-NKPAG-LVKLNPA
Zm  YITTLNVFQAYTLKRIRDPNFKVTPQPPLSKEFADE-NKPAG-LVKLNPA
```

```
         950       960
Ah  SEYPPGLEDTLILTMKGIAAGMQNTG
Bs  SEYAPGLEDTLILTMKGIAAGMQNTG
Ft  SEYAPGLEDTLILTMKGIAAGMQNTG
Ec  SEYGPGLEDTLILTMKGIAAGMQNTG
Sb  ERVPPGLEDTLILTMKGIAAGMQNTG
Si  SEYGPGLEDTLILTMKGIAAGMQNTG
So  SEYPPGLEDTLILTMKGIAAGMQNTG
Zm  SEYPPGLEDTLILTMKGIAAGMQNTG
```

1.2 Multiple Sequence Alignment of PPDK

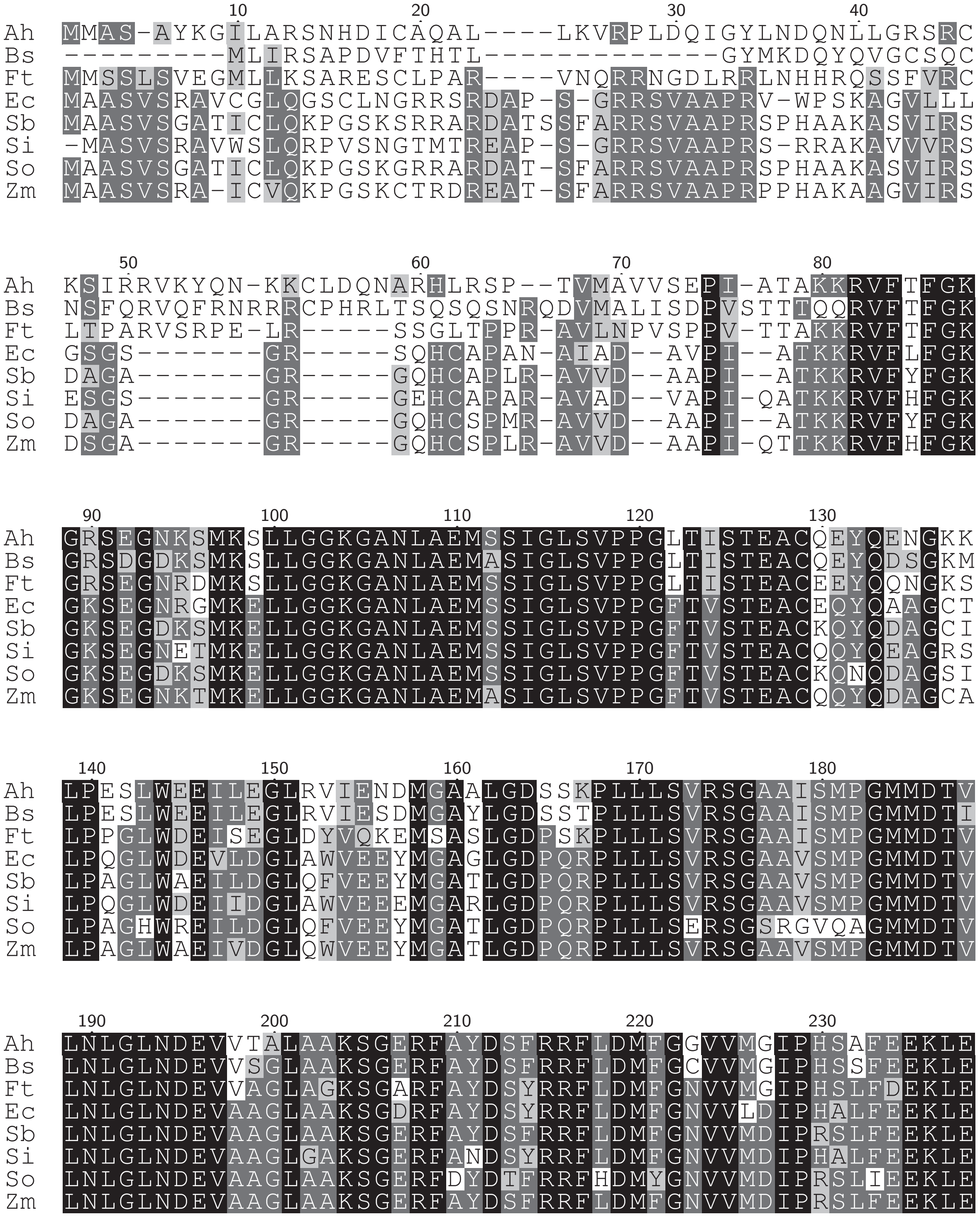

```
    240       250       260       270       280
Ah  KLKEEKGVKLDTELTASDLKELAEQYKNVYLETTGEAFPSSPLKQLQLAV
Bs  KLKQMKGVKLDTELTASDLKELAEQYKNVYIETKGEVFPADPKKQLQLAV
Ft  QMKAEKGIHLDTDLTAADLKDLVEKYKNVYVEAKGEKFPTDPKKQLELAV
Ec  HMKEAKGLKNDTDLTASDLKELVEQYKDVYVEAKGEPFPSDPKKQLELSV
Sb  HMKESKGVKNDTDLTAADLKELVGQYKEVYLTAKGEPFPSDPKKQLELAV
Si  AMKEAKGVKNDNDLTASDLKELVAQYKEVYVEAKGEPFPSDPKKQLELAV
So  HMKESKGVKNDTDLTAADLKELVGQYKEVYLTAKGEPFPSDPKKQLELAV
Zm  HMKESKGLKNDTDLTASDLKELVGQYKEVYLSAKGEPFPSDPKKQLELAV

    290       300       310       320       330
Ah  EAVFESWDSPRANKYRSINQISGLKGAAVNIQSMVFGNMGNTSGTGVLFT
Bs  QAVFDSWDSPRAVKYRSINQISGLKGTAVNIQSMVFGNMGNTSGTGVLFT
Ft  NAVFDSWDSPRANKYRSINQITGLKGTAVNIQSMVFGNMGNTSGTGVLFT
Ec  LAVFNSWDSPRAKKYRSINQITGLRGTAVNVQCMVFGNMGKTSGTGVLFT
Sb  RAVFNSWESPRAKKYRSINQITGLVGTAVNVQSMVFGNMGNTSGTGVLFT
Si  LAVFNSWDSPRANKYRSINQITGLKGTAVNVQSMVFGNMGDTSGTGVLFT
So  RAVFNSWESPRAKKYRSINQITGLVGTAVNVQSMVFGNMGNTSGTGVLFT
Zm  LAVFNSWESPRAKKYRSINQITGLRGTAVNVQCMVFGNMGNTSGTGVLFT

    340       350       360       370       380
Ah  RNPSTGEKKLYGEFLINAQGEDVVAGIRTPEDLGAMERCMPEAYKELVEN
Bs  RNPNTGERKLYGEFLINAQGEDVVAGIRTPEDLDTMKSCMPEAYTELVQN
Ft  RNPSTGEKKLYGEFLINAQGEDVVAGIRTPEDLGTMETCMPEAYKELVEN
Ec  RNPSTGEKKLYGEFLVNAQGEDVVAGIRTPEDLDAMKDCMPQAFEELVEN
Sb  RNPNTGEKKLYGEFLINAQGEDVVAGIRTPEDLDAMKDVMPQAYEELVEN
Si  RNPSTGEKKLYGEFLVNAQGEDVVAGIRTPEDLDAMKAQMPDAYVELVEN
So  RNPNTGEKKLYGEFPINAQGEDVVAGIRTPEDLDAMKDVMPQAYEELVEN
Zm  RNPNTGEKKLYGEFLVNAQGEDVVAGIRTPEDLDAMKNLMPQAYDELVEN

    390       400       410       420       430
Ah  CEILEQHYKDMQDIEFTVQENRLWMLQCRSGKRTGKGAVKIAVDLVNEGI
Bs  CEILEQHYKDMMDIEFTVQENRLWMLQCRSGKRTGKGAVKIAIDMVNEGK
Ft  CEILERHYKDMMDIEFTVQENRLWMLQCRTGKRTGKGAVRIAVDMVNEGL
Ec  CNLLEGHYKDLMDIEFTVQENRLWMLQCRSGKRTGKGAVKIAVDVVNEGL
Sb  CNILESHYKEMQDIEFTVQGNRLWMLQCRTGKRTGAGAVKIAVDMVSEGL
Si  CKILESHYKEMMDIEFTVQENRLWMLQCRSGKRTGQGAVKIAVDMVNEGL
So  CNILESHYKEMQDMEFTVQENRLWMLQCRTGKQTGTGAVKIAVDMVSEGL
Zm  CNILESHYKEMQDIEFTVQENRLWMLQCRTGKRTGKSAVKIAVDMVNEGL

    440       450       460       470       480
Ah  VDTNTAVKMVEPQHLDQLLHPQFEDPSAYKDKVIATGLPASPGAAVGQIV
Bs  INSRTAIKMVEPQHLDQLLHPQFEDASAYKERVITSGLPASPGAAVGQIV
Ft  IDTRTAIKRVETQHLDQLLHPQFEDPSAYKSHVVATGLPASPGAAVGQVC
Ec  VDRHAAIKMVEPGHLDQLLHPQFENPSAYKDQVIATGLPASPGAAVGQIV
Sb  VERRQAIKMVEPGHLDQLLHPQFENPALYKDKVIATGLPASPGAAVGQIV
Si  VERRQAIKMVEPGHLDHLLHPQFENPSAYKDQVIATGLPASPGAAVGQIV
So  AERRQAIKMVEPGHLDQLLHPQFENPAAYKDQVIATGLPASPGAAVGQIV
Zm  VEPRSAIKMVEPGHLDQLLHPQFENPSAYKDQVIATGLPASPGAAVGQVV

    490       500       510       520       530
Ah  FSAEDAEAWHAQGKSVILVRTETSPEDVGGMHVAAGILTARGGITSHAAV
Bs  FSAYDAEAWHAQGKSAILVRNETSPEDVGGMHAAAGILTARGGMTSHAAV
Ft  FSAEDAETWHAQGKSAILVRTETSPEDVGGMHAAAGILTARGGMTSHAAV
Ec  FTAEDAEAWHAQGKAAILVRTETSPEDVGGMHAAAGILTARGGMTSHAAV
Sb  FTAEDAEAWHAQGKAAILVRAETSPEDVGGMHAAAGILTERGGMTSHAAV
Si  FTAEDAEAWHAQGKAAILVRTETSPEDVGGMHAAVGILTARGGMTSHAAV
So  STAEDAEAWHAQGKAAILVRAETSPEDVGGMHAAAGILTERGGMTSHAAV
Zm  FTAEDAEAWHSQGKAAILVRAETSPEDVGGMHAAVGILTERGGMTSHAAV
```

```
    540       550       560       570       580
Ah  VARGWGKCCVSGCSEIQVNDAKKVVTIGNNVLAEGDWLSLNGTTGEVILG
Bs  VARGWGKCCVSGCSEIKVNETNKSLVVGNNVLTEGDWLSLNGSTGEVILG
Ft  VARGWGKCCVSGCADIRVNDDMKIFTIGDRVIKEGDWLSLNGTTGEVILG
Ec  VARGWGKCCVSGCASVRVNDAEKVVAIGDKVLQEGDWLSLNGSTGEVVLG
Sb  VARGWGKCCVSGCSAIRVNDAEKTVAIGDHVLSEGEWLSLNGSTGEVILG
Si  VARGWGKCCVSGCSSVLVNDAEKSVVIGDKVMHEGEWLSLNGSTGEVIVG
So  VARGWGKCCVSGCSAIRVNDAEKTVAIGDHVLSEGEWISLNGSTGEVILG
Zm  VARWWGKCCVSGCSGIRVNDAEKLVTIGSHVLREGEWLSLNGSTGEVILG

    590       600       610       620       630
Ah  KEPLAPPALSGDLEVFMSWADNLRRLKVMANADTPEDALTARNNGAEGIG
Bs  KEPLSPPALSGDLETIMSWTDAVRRLKVMANADTPEDALAARNNGAEGIG
Ft  KQLLAPPAMSNDLEIFMSWADQARRLKVMANADTPNDALTARNNGAQGIG
Ec  KQPLSPPALSGDLGIFMSWVDEVRKLKVLANADTPEDALAARNNGAEGIG
Sb  KQPLSPPALSGDLGTFMSWVDDVRKLKVLANADTPGDALAARNNGAQGIG
Si  KQPLSPPVLSGDLGTFMSWVDEVRQLKVLANADTPEDALTARNNGAEGIG
So  KQPLSPPSLSGDLGTFMSWVDEVRKLKVLANADTPEDALAARNNGAQGIG
Zm  KQPLSPPALSGDLGTFMAWVDDVRKLKVLANADTPDDALTARNNGAQGIG

    640       650       660       670       680
Ah  LCRTEHMFFASDDRIKTVRKMIMAVTPEQRKAALDQLLPYQRSDFEGIFR
Bs  LCRTEHMFFASDDRIKAVRKMIMAVTPEQRKAALDQLLPYQRSDFEGIFR
Ft  LCRTEHMFFASDERIKAVRKMIMAVTPEQRKVALDLLLPYQRSDFEGIFR
Ec  LCRTEHMFFASDGRIKAVRQMIMAPTLELRQKALDRLLPYQRSDFEGIFR
Sb  LCRTEHMFFASDERIKAVRQMIMAPTVELRQQALDRLLPYQRSDFEGIFR
Si  LCRTEHMFFASDERIKAVRQMIMAPTLELRQKALDRLLPYQRSDFEGIFR
So  LCRTEHMFFASDERIKAVRQMIMAPTVELRQQALDRLLPYQRSDFEGIFR
Zm  LCRTEHMFFASDERIKAVRQMIMAPTLELRQQALDRLLTYQRSDFEGIFR

    690       700       710       720       730
Ah  AMDGLPVTIRLLDPPLHEFPPEGDLDEIVKDLASEVGMTEDEVYSRIEKL
Bs  AMDGLPVTIRLLDPPLHEFLPEGDLQQIMNELASETGITEDEVYSRVEKL
Ft  AMDGLPVTIRLLDPPLHEFLPEGDLEHIVNELAVDTGMSADEIYSKIENL
Ec  AMDGLPVTIRLLDPPLHEFLPEGNVDEIVRELCAETGSNEEEALARIEKL
Sb  AMDGLSVTIRLLDPPLHEFLPEGNVEEIVRELCAETGANEEEALERVEKL
Si  AMDGLSVTIRLLDPPLHEFLPDGNVEDIVRELCSETGANQEEALARIESL
So  AMDGLSVTIRLLDPPLHEFLPEGNVEEIVRELCAETGANEEEALERVEKL
Zm  AMDGLPVTIRLLDHPSYEFLPEGNIEDIVSELCAETGANQEDALARIEKL

    740       750       760       770       780
Ah  SEVNPMLGFRGCRLGISYPELTEMQARAIFQAAVSMTNQGIKVLPEIMVP
Bs  SEVNPMLGFRGCRLGISYPELTEMQARAVFQAAVSMTNQGITVLPEIMVP
Ft  SEVNPMLGFRGCRLGISYPELTEMQVRAIFQAAVSMTNQGVTVIPEIMVP
Ec  SEVNPMLGFRGCRLGISYPELTEMQARAIFEAAITMTKPGCSSFSRDNVP
Sb  AEVNPMLGFRGCRLGISYPELTEMQARAIFEAAIAMSNQGVEVFPEIMVP
Si  SEVNPMLGFRGCRLGISYPELTEMQARAIFEAAIAMTNQGVQVFPEIMVP
So  AEVNPMLGFRGCRLGISYPELTEMQARAIFEAAIAMSNQGVEVFPEIMVP
Zm  SEVNPMLGFRGCRLGISYPELTEMQARAIFEAAIAMTNQGVQVFPEIMVP

    790       800       810       820       830
Ah  LVGTPQELSHQMGVIRDVASKVFSETGTTLTFKVGTMIEIPRAALIADEI
Bs  LVGTPQELGHQVNLIRSVATKVFSEMGSSVRYKVGTMIEIPRAALVADEI
Ft  LVGTPQELRHQISVIRGVAANVFAEMGVTLEYKVGTMIEIPRAALIAEEI
Ec  LVGTPQELGHQVAVIREIANTVFTAMGKTISYKIGTMIEIPRAALVADEI
Sb  LVGTPQELGHQVNVIKQTAEKVFANAGKTIGYKIGTMIEIPRAALVADQI
Si  LVGTPQELGNQVALIRETANKVFAALGKTIDYKIGTMIEIPRAALVADEI
So  LVGLPQELGHQVNVIKQVAEKVFTSMGKTIGYKIGTMIEIPRAALVADQI
Zm  LVGTPQELGHQVTLIRQVAEKVFANVGKTIGYKVGTMIEIPRAALVADEI
```

```
    840       850       860       870       880
Ah  AKEAEFFSFGTNDLTQMTFGYSRDDVGKFLPIYMAQGILQTDPFEVLDQK
Bs  AVEADFFSFGTNDLTQMTFGYSRDDVGKFLPKYLSNGILQADPFEVLDQK
Ft  GKEADFFSFGTNDLTQMTFGYSRDDVGKFLQIYLAQGILQHDPFEVIDQK
Ec  AEQAEFFSFGTNDLTQMTFGYSRDDVGKFLPIYLSQGILQHDPFEVLDQR
Sb  AEQAEFFSFGTNDLTQMTFGYSRDGVGKFIPIYLAQGILQHDPFEVLDQR
Si  AEQAEFFSFGTNDLTQMTFGYSRDDVGKFLPIYLSQGILQHDPFEVLDQR
So  AEQAEFFSFGTNDLTQMTFGYSRDDVGKFIPIYLAQGILQHDPFEVLDQR
Zm  AEQAEFFSFGTNDLTQMTFGYSRDDVGKFIPVHLAQGILQHDPFEVLDQR

    890       900       910       920       930
Ah  GVGQLIKHATEKGRASRPSLKVGICGEHGGEPSSVAFFAEAGLDYVSCSP
Bs  GVGQLIKLATEKGRAAKPSLKVGICGEHGGEPSSVAFFAEAGLDYVSCSP
Ft  GVGQLIKMATEKGRAANPSLKVGICGEHGGEPSSVAFFDGVGLDYVSCSP
Ec  GVGELVKFATERGRKTRPNLKVGICGEHGGEPLSVAFFAKTGLDYVSCSP
Sb  GVGELVKFATERGRQTRPNLKVGICGEHGGEPSSVAFFAKVGLDYVSCSP
Si  GVGELVKFATERGRKARPNLKVGICGEHGGEPSSVAFFAKSGLDYVSCSP
So  GVGELVKFATERGRQTRPNLKVGICGEHGGEPSSVAFFAKAGLDYVSCSP
Zm  GVGELVKFATERGRKARPNLKVGICGEHGGEPSSVAFFAKAGLDFVSCSP

    940       950
Ah  FRVPIARLAAAQVAV
Bs  FRVPIARLAAAQVAV
Ft  FRVPIARLAAAQVIV
Ec  FRVPIARLAADQVLV
Sb  FRVPIARLAAAQVLV
Si  FRVPIARLAAAQVLV
So  FRVPIARLAAAQVLV
Zm  FRVPIARLAAAQVLV
```
